# A prospective natural history study of Krabbe disease in a patient cohort with onset between 6 months and 3 years of life

**DOI:** 10.1186/s13023-018-0872-9

**Published:** 2018-08-09

**Authors:** Nicholas Bascou, Anthony DeRenzo, Michele D. Poe, Maria L. Escolar

**Affiliations:** Program for the Study of Neurodevelopment in Rare Disorders, Department of Pediatrics, Children’s Hospital of Pittsburgh of University of Pittsburgh Medical Center, 4401 Penn Avenue, Pittsburgh, PA 15224 USA

**Keywords:** Krabbe disease, Globoid cell leukodystrophy, Late-infantile, Infantile, Natural history, Newborn screening

## Abstract

**Background:**

Krabbe disease is a rare neurodegenerative disorder caused by a deficiency in the lysosomal enzyme galactocerebrosidase. Patients with Krabbe disease present with a variable disease course depending on their age of onset. The purpose of this prospective cohort study was to characterize the natural progression of Krabbe disease in a large group of patients with disease onset between 6 and 36 months of life who were evaluated with a standardized protocol.

**Methods:**

All patients with Krabbe disease who had onset between 6 and 36 months of age and were prospectively evaluated between 2000 to 2017 were included. Standardized neurodevelopmental, physical, and neurological examinations were performed. Other assessments included neuroradiologic and neurophysiologic tests, enzyme level, cerebrospinal fluid analysis, and *GALC* pathogenic variants when available. Descriptive statistics were used for analysis. Survival curve was estimated using the Kaplan–Meier method.

**Results:**

Thirty-five patients (26 boys, 9 girls) with disease onset between 6 and 36 months of age were evaluated. Median age at symptom onset was 11.5 months, with a median delay of 3.5 months between onset of symptoms and diagnosis. Of the 32 symptomatic patients, 23 presented with initial signs or symptoms of disease between 6 and 12 months of life; nine presented after 12 months. The most common initial signs and symptoms were loss of acquired developmental milestones, irritability, abnormal gait, motor delay, and abnormal muscle tone. The most common magnetic resonance imaging abnormality was increased T2 signal in the periventricular white matter. Nerve conduction velocity results were abnormal for 21 of 24 patients. Patients with onset after 12 months had less peripheral nerve involvement and slower disease progression. Abnormal cerebrospinal fluid protein levels were obtained for 13 of 16 symptomatic children. Protein levels were normal in all asymptomatic children.

**Conclusions:**

Based on our findings, we propose reclassifying the group of patients with onset ≤12 months as infantile and the > 12 month group as late-infantile. Patients with onset > 12 months are more likely to benefit from hematopoietic stem cell transplantation. The proposed change in classifications will allow physicians to improve their ability to recognize and diagnose patients and more precisely assess potential treatment effects after transplantation.

## Background

Krabbe disease, also known as globoid cell leukodystrophy, is a rare autosomal recessive metabolic disorder characterized by the deficiency of galactocerebrosidase (GALC), a lysosomal enzyme responsible for the hydrolysis of psychosine and galactosylceramide. The accumulation of psychosine is toxic to oligodendrocytes and Schwann cells and the failure to digest galactosylceramide leads to the formation of multi-nucleated globoid cells, causing severe demyelination, axonopathy, and neuronal death [1–4]. The degradation of the central and peripheral nervous systems clinically manifests as progressive neurodegeneration, spasticity, irritability, loss of vision and hearing, seizures, and premature death [5–8].

The incidence of Krabbe disease has been estimated as 1 in 100,000 live births [3]. The disease is typically divided into four subgroups based on age at symptom onset: early-infantile (birth–5 months), late-infantile (6–36 months), juvenile (37 months–16 years), and adult (> 16 years). However, there is some debate among experts regarding the age range for the late-infantile, juvenile, and adult phenotypes. The early-infantile form is the most common and rapidly progressing form of the disease [9–11]. Symptoms of the early-infantile phenotype include irritability, regression of psychomotor development, feeding difficulties and, as the disease progresses, hypertonicity, seizures, loss of vision and hearing, and early death [9–11]. The late-infantile phenotype, which accounts for 20–30% of the infantile cases, shows greater variability in clinical presentation. Symptoms of the late-infantile phenotype include psychomotor regression, ataxia, irritability and loss of vision [12–14]. The juvenile phenotype is characterized by loss of vision and psychomotor regression. Patients with the adult phenotype may have a normal life span but will slowly develop progressive spastic paraparesis or gait abnormalities [10, 14, 15]. Although more than 200 pathogenic variants of the *GALC* gene have been reported in the Human Gene Database, only a limited number of genotype-phenotype relationships have been established [14]. For example, at least 86 infantile pathogenic variants have been identified; however, for many of the 86 variants, there is no report as to whether they correlate specifically with the early-infantile or late-infantile phenotype [12, 15, 16].

To date, few natural history studies describing the clinical and biochemical characteristics of Krabbe disease have been published and the majority are based on retrospective and nonstandardized data. The first case series describing the infantile phenotypes was published in 1916 by the Danish clinician Knud Krabbe [17]. In 1969, the first large cohort of patients was described by Hagberg et al. [18], which described 32 Krabbe patients with early-infantile onset. Although more recent reports by Alodsari et al. [19] and Husain et al. [20] address neurophysiological and neuroradiological findings in Krabbe patients, neither discussed other clinical data and each included only 6 patients with onset after 6 months of age. Conversely, a recent retrospective study by Zhao et al. [14] reported on the biochemical, genetic, and brief clinical descriptions of 22 Chinese patients, including 10 with late-infantile onset, but did not provide results of neurophysiological or neuroradiological testing. Other publications attempting to provide a more comprehensive description of the disease’s natural history have relied on registry data; however, because registries are not based on a standardized clinical protocol and have less stringent data collection methods than prospective studies, they lack consistency in patient evaluations and are inherently subject to missing or inaccurately reported data [10, 11, 13]. For example, of the 12 late-infantile patients included in Duffner et al. [13], only 3 had results of auditory brainstem responses (ABR) or nerve conduction velocity (NCV) testing. Thus, there is an overwhelming need for prospective natural history studies that report specifically on phenotypes with onset after 6 months of age.

The only treatment currently available for Krabbe disease is hematopoietic stem cell transplantation (HSCT), which can favorably alter the natural course of the infantile subtypes if performed early enough in the disease progression [21–24]. Mandated newborn screening (NBS) programs for Krabbe disease have been implemented in several states throughout the U.S. and are crucial in early diagnosis and treatment. At risk infants identified through NBS have GALC activity levels < 0.50 nmol/hour/mg of protein. Depending on the enzyme activity level, infants are classified as being at high or moderate risk of developing disease. Some states also perform genetic analysis and measure psychosine levels to determine which individuals are at risk of developing the early-infantile form of the disease [25–28]. Although psychosine levels in dry bloods spots (DBS) have been shown to function as an effective predictor of disease progression in early-infantile Krabbe patients, there is less data in the literature describing correlations between psychosine levels and later onset phenotypes [29]. As a result of our lack of knowledge regarding correlations between genotype, GALC activity, psychosine levels, and disease progression, many newborns categorized as high risk require continued monitoring so they can be immediately evaluated for HSCT eligibility before they become too advanced to benefit from treatment. Thus, until precise biochemical and genotype-phenotype correlations can be established, natural history data will function as the main instrument in monitoring individuals who will develop disease following a positive NBS screening. In addition, natural history studies will provide the predominate means for evaluating the efficacy of newly development treatments, such as gene therapy and enzyme replacement therapy [30, 31].

Given the gaps in our knowledge, the intention of our study was to longitudinally describe the physical characteristics, signs, symptoms, and neurodevelopmental involvement in children diagnosed with Krabbe disease who had onset between 6 and 36 months of age. The patients were evaluated at a single site and followed throughout the course of their disease, allowing for internal consistency between the observations. Multiple standardized tests in all areas of development were performed using a prospectively designed protocol. Growth parameters were measured at every visit, and brain magnetic resonance imaging (MRI) and other neurophysiological analyses were carried out at baseline and longitudinally, when appropriate. Altogether, this is the largest and most comprehensive prospective study of Krabbe patients with onset between 6 and 36 months of age.

## Methods

### Subjects

This prospective cohort study included patients diagnosed with Krabbe disease who were evaluated at the Program for the Study of Neurodevelopment in Rare Disorders (NDRD) between January 2000 and September 2017 and had disease onset between 6 and 36 months of life. Diagnoses were made by measuring GALC activity in white blood cells or fibroblasts, done at the Lysosomal Diseases Testing Laboratory at Jefferson Medical College, and were confirmed by genetic analysis. However, since genetic analysis was not used consistently for diagnosis until approximately 2009, most patients diagnosed prior to 2009 lack genetic data. The patients were referred to the NDRD clinic for management of their symptoms and to assess their eligibility for treatment with HSCT.

### Neurodevelopmental evaluations

Children were evaluated following a comprehensive protocol of standardized testing designed by a multidisciplinary team for longitudinal follow-up at a single site [32]. At each clinic visit, a team of neurodevelopmental pediatricians, neurophysiologists, speech pathologists, audiologists, physical therapists, and psychometricians evaluated the patient for approximately 4–6 h. The neurodevelopmental tests included a physical and neurological exam for evaluation of signs and symptoms of disease, growth, mobility, adaptive behavior, cognitive behavior, physical characteristics, sensory function, and speech and language skills. Tests used to assess developmental function included the Mullen Scales of Early Learning, Peabody Developmental Motor Scales, Gross Motor Function Measure, Vineland Adaptive Motor Scales, and Scales of Independent Behavior-Revised [32–37]. As part of the standardized protocol, parents completed a questionnaire that asked about birth history, early signs of disease, development, and behaviors, including emergence of independent-adaptive behaviors. Patient outcomes were compared to the norms of typically developing children [32]. All research was conducted with the approval of the institutional review boards (IRB) from the University of North Carolina (IRB-08-0237) and the University of Pittsburgh (IRB-PRO11050036).

### Neuroradiologic and neurophysiologic testing

Using a 3 Tesla scanner, sagittal T1 FLAIR and T2 SPACE, and axial dual echo T2 and proton-density weighted brain MRI were obtained. Axial bold, diffusion, and susceptibility images were also obtained. MRI scans were interpreted by an experienced neuroradiologist and evaluated for any abnormalities. NCV motor responses were measured in the peroneal, tibial, and ulnar nerves, and sensory responses were measured in the sural and median nerves. NCV responses were considered abnormal if they showed prolongation of distal and F-wave latencies, low amplitude, or no evoked response. Flash visual evoked potentials (VEP) were considered abnormal if the P100 wave was absent. ABR were considered abnormal if wave I–V interpeak latencies were prolonged or any of the obligate waveforms (I, III, or V) were absent.

### Cerebrospinal fluid (CSF) protein analysis

CSF was obtained by lumbar puncture while the patient was under general anesthesia for MRI or local anesthesia. Total CSF protein was determined by tandem mass spectrometry.

### Severity score index

Patients with onset between 9 and 12 months were split into two groups, those with a severe phenotype and those with a less severe phenotype; the two groups were compared in clinical variables. A severity index was developed to assess the markers’ ability to predict disease phenotype. The ten most relevant markers of disease were selected by a group of experts in Krabbe disease. Severity scores were generated by summing the total number of markers observed in each patient. The patient’s severity score was calculated at the time of their baseline evaluation.

### Statistical analysis

For patients who were lost to follow-up, the Social Security Death Index was queried to search for any deaths that occurred after the patient’s last evaluation (http://search.ancestry.com). Survival curves were estimated using the Kaplan–Meier method. Patients who received HSCT were included up to the day they began the HSCT protocol, at which point they were censored. All other surviving patients were censored on September 2, 2017. Clinical growth charts were created based on the published Centers for Disease Control growth charts [38]. Developmental growth charts were created by plotting the patient’s age-equivalent (AE) score against their actual age. AE scores are ideal for longitudinal analysis in neurodegenerative disorders, as they can be used to ascertain whether a child is gaining or losing skills over time [32]. To test for differences in developmental abilities between the patients with onset > 12 months, onset ≤12 months, and the population norms, the age of the child was subtracted from the AE score to create a norm centered value.

Using SAS 9.4, mixed regression models were fitted to test for group differences with the AE score, as the dependent variable, and group, age, and the group x age interaction as independent variables. To account for repeated evaluations, patient age was entered as a random variable. Differences between groups were tested by examining the group x age interaction for significance (*p* < 0.05). Group specific rates of development were calculated using post-estimation procedures. Differences between the group rate and normal development were then examined for significance.

## Results

### Patient characteristics

The 35 patients evaluated in this study included 26 boys and 9 girls between the ages of 2 and 84 months (2 black, 2 Asian, 30 white, and one not reported). Eleven children were evaluated longitudinally (median number of visits = 2, range = 2–8), and 24 were evaluated only once. For 15 children, only the baseline evaluation was available since these patients subsequently underwent HSCT. Nine children were lost to follow-up for reasons related to travel or financial difficulties. The average age at diagnosis was 17.8 months (median = 16, range = 0–39). Mean GALC activity was 0.048 nmol/h/mg protein (range = 0–0.29 nmol/mg/h protein; normal range = > 0.8 nmol/mg/h protein). The average delay between appearance of initial symptoms and diagnosis of Krabbe disease was approximately 4.6 months (median = 3.5, range = 0–21). Three patients were asymptomatic at the time of diagnosis and were diagnosed because of their family history.

### Neonatal history

Sixteen of the children developed neonatal difficulties; eight presented with multiple difficulties. Difficulties included jaundice requiring phototherapy (*n* = 8), vomiting (*n* = 4), feeding difficulties (*n* = 2), colic (*n* = 2), occasional coughing (*n* = 1), gastroesophageal reflux (*n* = 1), respiratory distress (*n* = 3), fetal distress due to meconium (*n* = 1), severe hypoglycemia (*n* = 1), lethargy (*n* = 1), and low temperature (*n* = 1).

### Initial signs and symptoms

Initial signs and symptoms of disease were defined as a change that caused parental and/or physician concern. Because 3 patients were diagnosed due to family history and immediately underwent HSCT while still asymptomatic, data on initial signs and symptoms was only available for 32 of the 35 patients. For the 32 symptomatic patients, the most common initial signs and symptoms were loss of acquired developmental milestones (*n* = 13, 41%), irritability (*n* = 12, 38%), abnormal gait (*n* = 7, 22%), motor delay (*n* = 5, 16%), abnormal muscle tone (*n* = 4, 13%), and slurred speech (*n* = 3, 9%). Less common initial signs and symptoms were poor feeding (*n* = 2, 6%), loss of vision (*n* = 2, 6%), macrocephaly (*n* = 1, 3%), and decreased arm movements (*n* = 1, 3%). Of the 32 symptomatic patients in the study, 23 presented with initial signs or symptoms of disease between 6 and 12 months, and 9 presented after 12 months of life. The majority of patients with onset after 12 months of age initially presented with abnormal gait (*n* = 6; 67%). However, only 1 patient with onset before 12 months learned to walk, and they most often presented initially with loss of early developmental milestones (*n* = 13; 52%) (i.e. cooing, head control, independent sitting) (Table 1).

**Table 1:** Initial signs and symptoms of disease

Initial Signs and Symptoms	All Ages n/N (%)	Age of Onset	
		≤12 Months n/N (%)	> 12 Months n/N (%)
Loss of developmental milestones	13/32 (41)	12/23 (52)	1/9 (11)
Irritability	12/32 (38)	8/23 (35)	4/9 (44)
Abnormal gait	7/32 (22)	1/23 (4)	6/9 (67)
Motor delay	5/32 (16)	5/23 (22)	0/9 (0)
Abnormal muscle tone	4/32 (13)	4/23 (17)	0/9 (0)
Slurred speech	3/32 (9)	1/23 (4)	2/9 (22)
Loss of vision	2/32 (6)	0/23 (0)	2/9 (22)
Abnormal arm movements	1/32 (3)	1/23 (4)	0/9 (0)
Macrocephaly	1/32 (3)	1/23 (4)	0/9 (0)

Displays number and percentage of children who presented with each sign or symptom as an initial indicator of disease. Children are separated into age groups based on age at the onset of first symptoms

### Feeding and other gastrointestinal problems

**Fig. 1:**
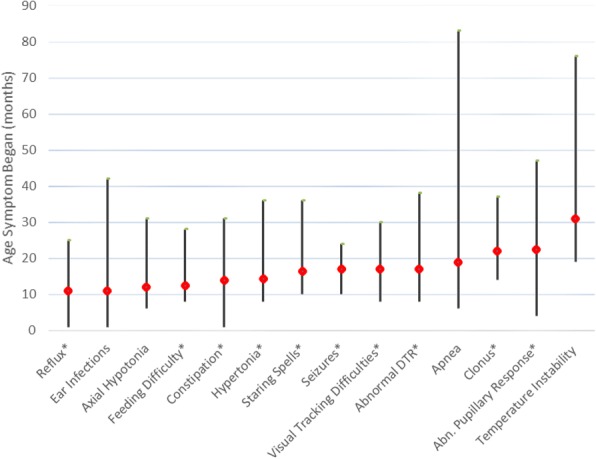
Ages at which common symptoms appear in children with Krabbe disease. The red diamond represents the median age at which the symptom began. The lines show the minimum and maximum ages that the symptom began. Symptoms that were used in creating the severity index are designated with asterisks

Feeding difficulties were present in 23 children (72%), with a median age of onset at 12.5 months (range = 8–28) (Fig. 1, Table 2). Four children (13%) had either a history of or current difficulty with latching onto the breast/bottle. Seventeen children (53%) had symptoms of gastroesophageal reflux (median age at onset = 11 months, range = 1–25). Twenty-two children (69%) had symptoms of constipation (median age at onset = 14 months, range = 1–31). Thirteen children (41%) were reported by parents to have slow weight gain, and 9 children (28%) were diagnosed with failure to thrive. In 6 of the children with feeding difficulties, a gastrostomy tube had been placed prior to their baseline evaluation. After the initial evaluation, gastrostomy tube placement was recommended for 15 more children.

**Table 2:** Signs and symptoms of diseases in respect to age of onset

Clinical Indicators of Disease Progression	All Ages n/N (%)	Age at Onset	
		≤12 Months n/N (%)	> 12 Months n/N (%)
Abnormal protective reflexes	29/32 (91)	23/23 (100)	6/9 (67)
Axial hypotonia	29/32 (91)	23/23(100)	6/9 (67)
Appendicular hypertonia	28/32 (88)	20/23 (87)	8/9 (89)
Abnormal deep tendon reflexes	22/29 (76)	17/22 (77)	5/7 (71)
Constipation	22/32 (69)	16/23 (70)	6/9 (77)
Feeding difficulty	23/32 (72)	20/23 (87)	3/9 (33)
Thumb clasping or hand fisting	19/32 (59)	18/23 (78)	1/9 (11)
Asymmetric tonic neck reflex	18/32 (56)	17/23 (74)	1/9 (11)
Babinski reflex	18/32 (56)	15/23 (65)	3/9 (33)
Abnormal pupillary response	18/32 (56)	13/23 (56)	5/9 (56)
Gastroesophageal reflux	17/32 (53)	15/23 (65)	2/9 (22)
Open mouth posture	15/32 (47)	13/23 (56)	2/9 (22)
Slow weight gain	13/32 (41)	11/23 (48)	2/9 (22)
Failure to thrive	9/32 (28)	9/23 (39)	0/9 (0)
Appendicular hypotonia	2/32 (6)	1/23 (4)	1/9 (11)

Displays common clinical indicators of disease progression observed by the physician or reported by parents at evaluation. Children are separated into age groups based on age at the onset of first symptoms

### Disease progression

Following onset, the earliest symptoms in this patient group were gastrointestinal reflux, ear infections, and axial hypotonia, followed by feeding difficulties, constipation, appendicular hypertonia, and staring episodes. As the disease progressed, visual difficulty, apneic episodes, seizures, and temperature instability became more common (Fig. 1).

#### Growth

**Fig. 2:**
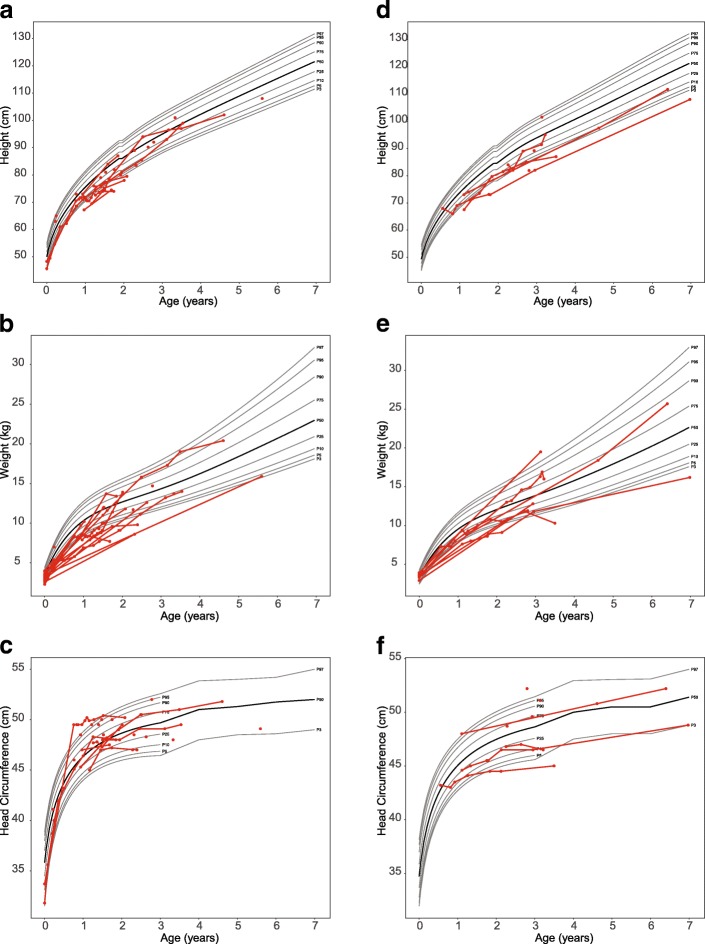
Height, weight, and head circumference of boys and girls with Krabbe disease. The x axis shows the patient's age in years and the y axis shows the height in centimeters. Each circle depicts an individual measurement; lines connecting circles show multiple measurements for an individual child. The gray lines represent standard growth curves (gray lines = 3rd, 5th, 10th, 25th, 50th, 75th, 90th, 95th, and 97th percentiles)

Most children had height and weight measurements below the 50th percentile. Five patients had a weight more than two standard deviations below the population mean during one or more visits, and 2 had a height more than two standard deviations below the population mean. In contrast, no patient had a head circumference measurement greater or lesser than two standard deviations from the mean (Fig. 2).

#### Survival

**Fig. 3:**
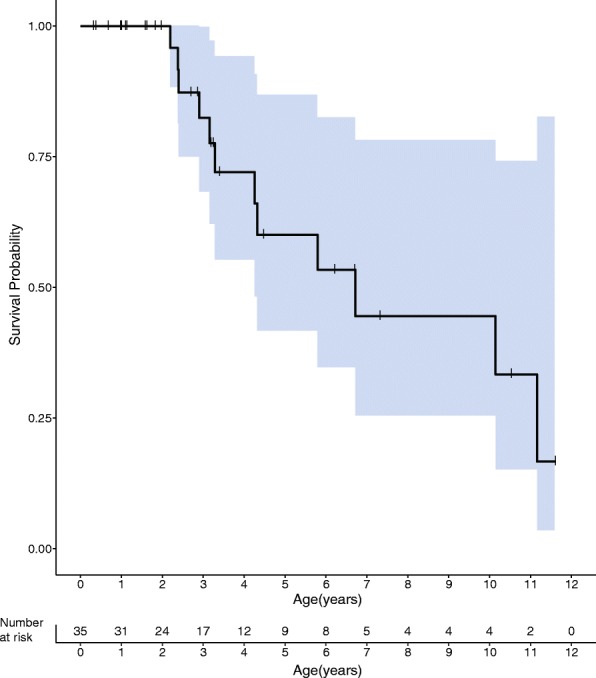
Kaplan–Meier curve of overall survival. The blue shaded area represents the 95% confidence interval. The overall median survival was 6.7 years. The x axis shows age in years and below the number of patients at risk for an event. The y axis probablity of survival

Twelve children (34%) died during the course of the study. Results of the Kaplan–Meier analysis indicate the median survival time is 6.72 years (95% Confidence Interval: 4.26, ∞) (Fig. 3).

#### Changes in muscle tone and reflexes

Twenty-nine of the 32 symptomatic children (91%) had axial hypotonia upon examination (median = 12 months, range = 6–31). The three symptomatic children who did not present with axial hypotonia did not become symptomatic until after 2 years of age. Two symptomatic children (6%) had appendicular hypotonia, and 28 symptomatic children (88%) had appendicular hypertonia. Age of onset for appendicular hypertonia was available for 26 children (median age = 14 months, range = 8–36). Twenty-nine symptomatic children (91%) showed abnormal protective reflexes upon examination. Clasped thumb or fisted hands were noted during examination in 19 symptomatic children (59%). Deep tendon reflexes (DTR) were abnormal in 22 of the 29 symptomatic children (76%) with available data (median age = 22 months, range = 8–38). DTR responses were not assessable for 3 children because of irritability or lack of cooperation.

#### Vision and hearing

Visual difficulties were apparent in 12 children. Median age at onset was 22 months (range = 12–35) (Fig. 1). Vision deterioration was observed in 9 of the 10 children longitudinally evaluated. Three children presented with cortical blindness. Of these 3, two experienced vision loss preceding changes in muscle tone, and one experienced vision loss following changes in muscle tone. The most common form of abnormal eye movement was disconjugate gaze (48%), followed by strabismus (14%) and nystagmus (11%). Eighteen of 32 symptomatic children (56%) had abnormal pupillary responses to light (Table 2). Eight of the 19 children (42%) for whom data on VEP were available had abnormal results, and 1 had inconclusive results due to lack of cooperation. One child with abnormal VEP was asymptomatic at the time of assessment.

Results of baseline hearing tests were available for 27 patients; none had hearing loss at the time of diagnosis. One child’s results were inconclusive because of a sinus infection. Recurring ear infections were reported in 11 symptomatic children (34%). Four of the children evaluated longitudinally had abnormal tympanometry results on subsequent exams (median age = 41 months, range = 29–67). ABR were abnormal in 20 of the 26 children (77%) who were tested. Two of the children with abnormal recordings were asymptomatic upon evaluation. The most common abnormality was prolongation in latencies that progressed from wave I to wave V (from the auditory nerve towards the brainstem).

### Neurodevelopmental skills

#### Developmental milestones

Developmental milestones achieved by symptomatic children included head control (*n* = 32), rolling over (*n* = 28), independent sitting (*n* = 24), crawling (*n* = 17), and independent walking (*n* = 9) (Table 3). Gait was abnormal in all children who learned to walk. Most children rapidly lost previously achieved milestones after disease onset.

**Table 3:** Achievement of developmental motor milestones

Milestones	Achieved, All Ages n/N (%)	Achieved, Onset ≤12 Months, n/N (%)	Achieved, Onset > 12 Months, n/N (%)
Roll over	28/32 (88)	19/23 (83)	9/9 (100)
Sit independently	24/32 (75)	15/23 (65)	9/9 (100)
Crawl	17/32 (53)	8/23 (35)	9/9 (100)
Walk independently	9/32 (28)	1/23 (4)	8/9 (89)

Displays the percentage of children who achieved developmental motor milestones. Children are separated into age groups based on age at the onset of first symptoms

#### Overall function

**Fig. 4:**
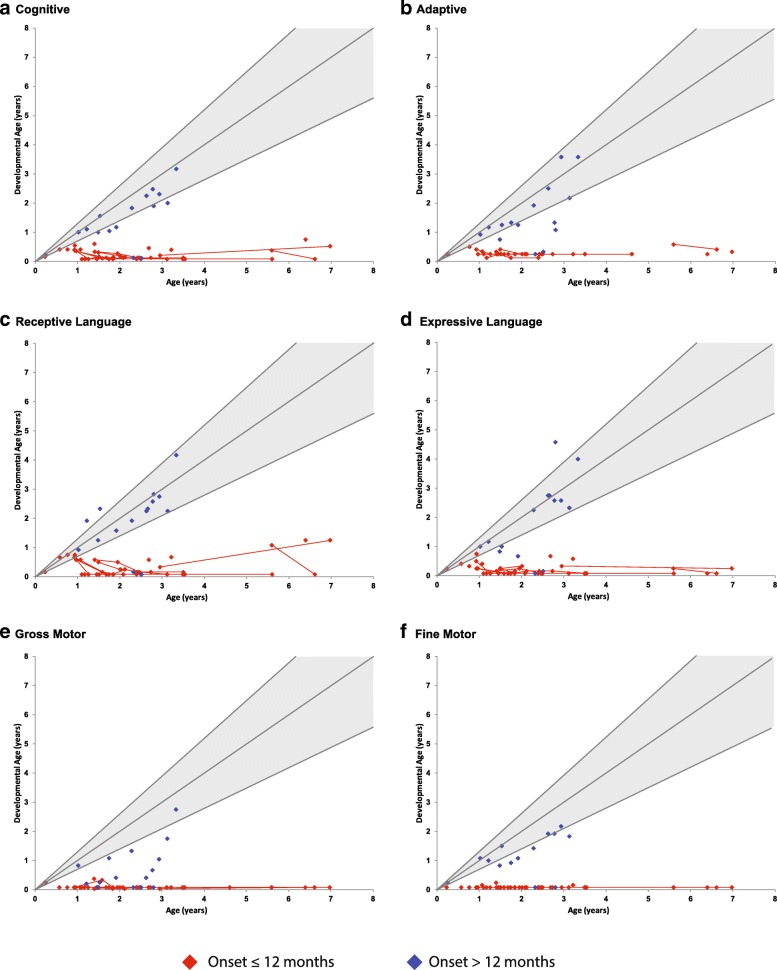
Developmental progression of children with Krabbe disease from birth to 8 years of age. Age-equivalent scores (i.e., developmental age) are graphed against actual age for (**a**) cognitive development, (**b**) adaptive behavior, (**c**) receptive language, (**d**) expressive language, (**e**) gross motor function, and (**f**) fine motor function to allow comparisons across tests and monitor development over time. The lines and diamonds represent individual patients, with red indicating patients with disease onset ≤12 months and blue indicating patients with onset > 12 months. The shaded gray area represents typical development with the lines representing the mean and approximate 95% interval for typically developing children

**Fig. 5:**
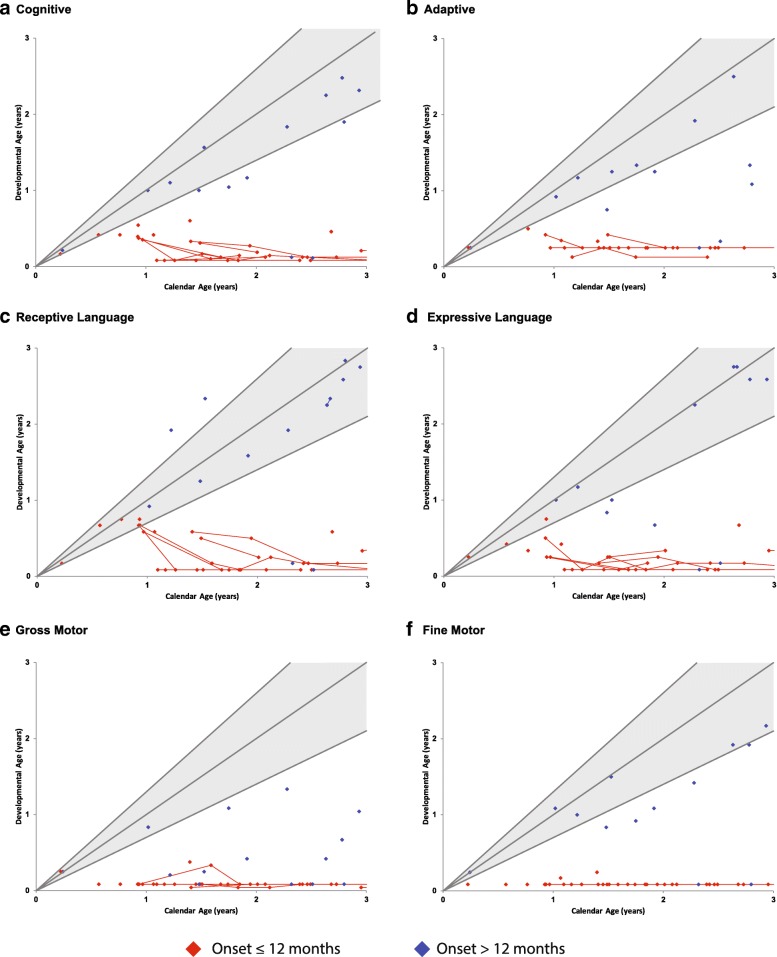
Close up of developmental progression of children with Krabbe disease, from birth to 3 years of age. Age-equivalent scores (i.e., developmental age) are graphed against actual age for (**a**) cognitive development, (**b**) adaptive behavior, (**c**) receptive language, (**d**) expressive language, (**e**) gross motor function, and (**f**) fine motor function to allow comparisons across tests and monitor development over time. The lines and diamonds represent individual patients, with red indicating patients with disease onset ≤12 months and blue indicating patients with onset > 12 months. The shaded gray area represents typical development with the lines representing the mean and approximate 95% interval for typically developing children

Cognitive function, adaptive behavior, expressive and receptive language, and motor development were evaluated for each patient. As a whole, patients in the study scored significantly lower than age-matched controls in all developmental domains other than expressive language. In addition, patients with onset after 12 months of age scored significantly higher than patients who presented before 12 months in cognitive ability (*p* < 0.001), expressive language (*p* < 0.001) and receptive language (*p* = 0.015), gross motor (*p* = 0.034), fine motor, (*p* < 0.001), and adaptive abilities (*p* < 0.001) (Fig. 4 & Fig. 5). However, the > 12 month group still performed significantly lower than the normal age-matched pediatric population in all developmental domains (*p* < 0.001) except receptive (*p* = 0.076) and expressive language (*p* = 0.521).

#### Cognitive function

Cognitive function was assessed using standardized protocols that tested the ability to listen, solve visual problems, and perform simple tasks. While some children regressed in development very quickly, others had developmental quotients between the 5th and 95th percentiles. However, all children evaluated longitudinally eventually fell below the 5th percentile in cognitive development by 40 months of age (Fig. 4a & Fig. 5a). Only 1 child acquired new cognitive skills after the baseline evaluation.

#### Language development

After the baseline evaluation, only 1 child gained receptive language skills, and all other children regressed in both receptive and expressive language skills (Fig. 4c, d & Fig. 5c, d). No gains in expressive language skills were observed following any patients baseline visit. For most patients, receptive language remained intact longer than expressive language.

#### Fine motor skills

All children evaluated longitudinally initially presented with severe fine motor delay/decline, and no patient gained fine motor skills in subsequent assessments. Although regression in fine motor skills typically began about the same time as gross motor regression, some children maintained fine motor skills slightly longer (Fig. 4f & Fig. 5f).

#### Gross motor skills

The only children who gained skills in gross motor development were those who had not yet experienced changes in muscle tone and were considered asymptomatic (*n* = 2) or minimally symptomatic (*n* = 1) (Fig. 4e & Fig. 5e).

#### Adaptive behavior

Adaptive behavior was significantly hindered by gross and fine motor symptoms. All children who were longitudinally evaluated showed delayed development in adaptive behavior at the initial evaluation and subsequently lost previously gained skills (Fig. 4b & Fig. 5b).

### Neuroradiologic and neurophysiologic testing

#### Magnetic resonance imaging (MRI)

MRIs were available for 34 of the 35 patients. Of these, all but 1 were abnormal. The single normal scan was from an asymptomatic child younger than 3 months. The most common abnormality was increased T2 signaling in the periventricular white matter, sometimes extending into the central and subcortical white matter. Twenty patients had abnormalities in the corticospinal tract, with T2 signaling specifically observed in the internal capsule for 9 patients. Other abnormalities were in the corpus callosum (*n* = 15), cerebellar white matter (*n* = 9), and corona radiata (*n* = 5).

#### Nerve conduction velocity

NCV results were available for 24 patients. Twenty-one of the 24 (88%) studies were abnormal. All 21 abnormal studies showed motor nerve involvement, and 13 results (62%) showed sensory nerve involvement. Seventeen of the studies (85%) showed motor F-wave abnormalities. Of the 21 abnormal results, 11 (52%) were considered severe, showing sensory, motor, and F-wave involvement; 7 (33%) were considered moderate because they showed at least two forms of involvement; and 3 (14%) were considered mild, for they showed only one form of involvement. One child with an abnormal result was asymptomatic at the time of evaluation.

#### Electroencephalography and seizure activity

Ten of the 32 symptomatic children (31%) had a history of clinical seizures. Onset data was available for 9 of these children (median age = 17 months, range = 10–24) (Fig. 1). Seizures increased in frequency as the disease progressed for 4 of the children. EEG results were available for 18 patients, of which 11 (61%) were abnormal. Abnormal background slowing was reported for 88% of patients, and abnormal spikes were reported in 63% of patients. One child with abnormal EEG results was asymptomatic at the time of evaluation. Fifteen symptomatic children (47%) experienced frequent staring episodes, which were not always captured as a seizure on EEG.

### Cerebrospinal fluid protein

CSF levels were available for 16 symptomatic children and 2 asymptomatic children. The median CSF protein level for the symptomatic cohort was 126 mg/dL (range = 27–214 mg/dL), with elevated levels detected in 13 children. CSF protein levels for the two asymptomatic children were within the normal range (35 and 55 mg/dL).

### Genetic analysis

Results of genetic analysis were available for 20 children (Table 4). Eight children were heterozygous for the 30 kb deletion. All 8 compound heterozygotes showed symptoms within their first year of life and all experienced the emergence of abnormal DTR’s (median age = 11 months, range = 9–32); however, there were no other commonalities in their disease progression that would suggest a correlation with the deletion.

**Table 4:** Genetic variants, GALC activity, and initial symptoms

Patient Number	Age of Onset (months)	Initial Symptoms	GALC Activity	Allele 1	Allele 2
1	6	Loss of developmental milestones	0.02	p.Thr529Met	30 kb deletion (exon 11–17)
2	6	Motor delay; Macrocephaly	0.06	30 kb deletion (exon 11–17)	p.Ile546Thr
3	6	Poor feeding	0	p.Gly57Ser; p.Ile562Thr	30 kb deletion (exon 11–17)
4	6	Irritability; Abnormal muscle tone	0	Unknown	Unknown
5	7	Motor delay	0.29	p.Gly284Ser	30 kb deletion (exon 11–17)
6	7	Loss of developmental milestones	0	p.Gly284Ser; p.Ile562Thr	p.Ile562Thr; p.Leu650Pro
7	7.5	Abnormal tone	0	p.Arg82Gly; p.Asp248Asn; p.Tyr319Cys	p.Ser303Phe; p.Ile562Thr
8	8	Loss of developmental milestones	0.04	p. Arg82Gly; p.Asp248Asn; p.Ala306Thr	p. Arg82Gly; p.Asp248Asn; p.Ala306Thr
9	8	Irritability; Abnormal muscle tone; Loss of developmental milestones	0.1	30 kb deletion (exon 11–17)	p.Thr529Met; p.Ile562Thr
10	8	Motor delay; Loss of developmental milestones	0.05	Unknown	Unknown
11	8	Loss of developmental milestones	0.13	Unknown	Unknown
12	9	Motor delay	0	30 kb deletion (exon 11–17)	p.Arg531Cys
13	9	Poor feeding	0.06	p.Ile128Lfsx27; p.Arg184Cys; p.Ile562Thr	p.Leu645Arg
14	10	Irritability	0.02	30 kb deletion (exon 11–17)	p.Leu325X
15	10	Loss of developmental milestones	0	Unknown	Unknown
16	10	Irritability; Loss of developmental milestones	0.05	Unknown	Unknown
17	11	Irritability; Loss of developmental milestones	0.2	p.Thr529Met	p.Thr529Met
18	12	Abnormal gait	0	p.Met117Val; p.Ile562Thr	p.Ser303Pro; p.Ile562Thr
19	12	Motor Delay	0.05	Unknown	Unknown
20	12	Irritability; Loss of developmental milestones	0.05	Unknown	Unknown
21	12	Irritability; Decreased movements; Loss of developmental milestones	0.05	Unknown	Unknown
22	12	Irritability; Abnormal muscle tone	0	Unknown	Unknown
23	12	Loss of developmental milestones	0.05	p.Thr529Met	p.Tyr567Ser
24	15	Abnormal gait	0	Unknown	Unknown
25	17	Loss of developmental milestones	0	p.Ala225Glu	p.Gly284Ser; p.Ile562Thr
26	24	Abnormal gait	0.12	p.Thr625Ala; Leu634Ser	p.Arg127Cys
27	24	Abnormal gait	0.08	Unknown	Unknown
28	25	Loss of vision; Irritability	0	Unknown	Unknown
29	25	Abnormal gait; Irritability	0.05	Unknown	Unknown
30	28	Abnormal gait; Slurred speech; Irritability	0.06	p.Arg127X; p.Ile562Thr	p.Gly284Ser; p.Ile562Thr
31	30	Loss of vision	0	p.Tyr319Cys	p.Tyr319Cys
32	31	Abnormal gait; Slurred speech	0.07	p.Met117Val; p.Ile562Thr	p.Ser303Phe p.Ile562Thr
33	Asymptomatic	N/A	0	p.Met117Val; p.Ile562Thr	p.Ser303Pro; p.Ile562Thr
34	Asymptomatic	N/A	0	Unknown	Unknown
35	Asymptomatic	N/A	0.08	Unknown	Unknown

Displays the initial symptoms, GALC activity (measured in nmol/mg/h protein), and available genetic data (*n* = 20) for each patient. All mutations are transcribed based on standardized nomenclature from the Human Genome Variation Society (HGVS) [39]. Multiple variants located on the same allele are separated by a semi-colon. Patient 33, 34, and 35 were diagnosed and transplanted while still asymptomatic due to a family history of Krabbe disease. Patient 33 is the younger sibling of patient 18 and patient 35 is the younger sibling of patient 19 and 24. The older sibling of patient 34 was not included in the study

### Severity score index

Ten clinical markers were identified as effective predictors for the severe, rapidly progressing phenotype when observed in patients who had onset between 9 and 12 months of age: reflux, constipation, feeding difficulty, tracking difficulty, abnormal pupillary response, staring spells, clonus, appendicular spasticity or hypertonia, seizures, and abnormal DTRs. Of the 12 patients with onset in this age range, 7 were classified as severe and 5 were classified as less severe. Clear differences in developmental abilities are delineated in Figs. 4 and 5, with the severe group scoring significantly higher on baseline evaluations. All children who scored a 6 or above on the severity index progressed rapidly following onset, failed to gain any developmental skills, and did not receive HSCT as per physician recommendation. Conversely, all 5 patients scoring ≤5 on the index progressed significantly slower, with 4 undergoing HSCT following their baseline evaluation. Notably, all patients in the severe group presented with feeding difficulties, clonus, and abnormal DTR’s by the time of their baseline clinical evaluation. For a comprehensive breakdown of the 12 patients’ clinical profiles with respect to the 10 markers, see Table 5.

**Table 5:** Severity score index for patients with onset between 9 and 12 months

Age of Onset	Age of Initial Evaluation	Rate of Disease Progression	Transplanted?	Reflux	Constipation	Feeding Difficulties	Tracking Difficulties	Abnormal Pupillary Response	Staring Episodes	Seizures	Clonus	Abnormal DTR
12	12	Slower	Yes	0	0	0	0	0	0	0	0	0
12	23	Slower	Yes	0	0	0	0	0	0	0	0	0
10	17	Slower	Yes	0	1	0	0	0	0	0	0	1
12	21	Slower	Yes	1	0	1	0	1	0	0	0	0
9	14	Slower	No	1	1	1	0	0	0	0	0	1
10	32	Rapid	No	0	0	1	0	1	1	0	1	1
10	13	Rapid	No	1	1	1	1	0	0	0	1	1
12	14	Rapid	No	1	1	1	0	1	0	1	1	1
12	38	Rapid	No	1	1	1	1	0	1	1	1	1
12	17	Rapid	No	0	1	1	1	1	1	1	1	1
11	35	Rapid	No	1	1	1	1	1	1	1	1	1
9	14	Rapid	No	1	1	1	1	1	1	1	1	1

Displays clinical data for the 12 patients with disease onset between 9 and 12 months of life. A 1 indicates that the patient presented with the clinical marker at their first evaluation and a 0 indicates that the patient did not present with the clinical marker at their first evaluation. Values in the total column represent the patient’s severity score. Patients scoring a 6 or above are expected to progress more rapidly than patients with a score of 0–5

## Discussion

We have prospectively evaluated a large cohort of patients with Krabbe disease evaluated at the Program for the Study of Neurodevelopment in Rare Disorders over a 17 year period. This is the first comprehensive natural history study of Krabbe disease in patients with onset between 6 and 36 months of life and represents the largest prospective study of patients with this disease. With this study, we were able to characterize common features of the disease and advance our systematic understanding of the phenotypic variability. For many patients in the study, irritability, abnormal DTR’s, feeding difficulties accompanied by axial hypotonia, constipation, and loss of protective reflexes functioned as early clinical markers of disease. In addition, many patients had ongoing problems with reflux (53%) and recurrent ear infections (34%), which frequently manifested early and preceded neuromuscular symptoms. As the disease progressed, patients often developed seizures, vision loss, apneic episodes, and signs of dysautonomia (i.e. temperature instability). Overall, phenotype varied significantly depending on the age of onset.

Looking further into the data, we were able to identify two distinct trajectories for patients with onset between 6 and 36 months, with the first accounting for most patients with onset between 6 and 12 months and the second accounting for all patients with onset between 13 and 36 months. Specifically, patients with onset prior to 12 months of age presented with a more severe phenotype and progressed more rapidly than patients with onset after 12 months. That is, most children who experienced onset before 12 months were delayed in developing early milestones such as the ability to roll over, sit, and crawl independently, and the overwhelming majority never learned to walk. In contrast, the majority of symptomatic children with later onset (> 12 months) developed motor milestones within the normal age range, with all but one developing the ability to walk (Table 3). Thus, whereas patients with onset ≤12 months most often presented initially with motor delay or rapid loss of acquired developmental milestones (cooing, head control, independent sitting), patients with onset > 12 months were typically diagnosed after a physician or parent noticed changes in gait, characterized by poor balance, ataxia, a wide base, and decreased trunk rotation. Other disparities in clinical presentation between the two age groups included feeding difficulties, gastroesophageal reflux, failure to thrive, slow weight gain, abnormal protective reflexes, clasped thumb and hand fisting, axial hypotonia, and asymmetric tonic neck reflexes, all of which were significantly more common in the ≤12 month onset group. In terms of developmental function, there were significant differences between the two groups in cognitive ability, receptive and expressive language, gross and fine motor abilities, and adaptive skills. Although one child with onset before 12 months showed increased cognitive skills at their second evaluation, the results for this child likely lack validity because the patient was fatigued during their baseline evaluation. It is also notable that the two patients with onset after 12 months who scored markedly low on baseline developmental testing were evaluated well after disease progression had begun.

Despite the overt differences emerging between the group of patients with onset before 12 months of age and the group of patients with onset after 12 months, there was some, albeit less, heterogeneity in the phenotype of patients within the 6–12 month cohort. As an example, all symptomatic patients with onset before 9 months of age had severe cognitive and motor impairment, experienced rapid disease progression, and failed to gain any functional skills following initial signs and symptoms of disease. Nevertheless, when we looked at the 12 symptomatic patients with onset between 9 and 12 months, we found that 7 exhibited the severe phenotype similar to the < 9 month group, and that 5 exhibited a less severe phenotype (characterized by normal or borderline normal cognition and markedly better adaptive and fine motor skills). Thus, these findings indicate that the phenotype for patients with onset before 9 months is highly predictable and the majority of patients with onset between 9 and 12 months have a severe phenotype similar to the < 9 month group. However, the findings also indicate a degree of variability in the 9–12 month cohort, which can lead to uncertainty when predicting the phenotype of a newly diagnosed patient with onset between 9 and 12 months. This is because the 9–12 months period is a critical window in the development of walking, in which corticospinal tract integrity is necessary to acquire an sufficient gross motor function. Therefore, this is a key time point in development for the patients manifesting severe disease versus a more mild form.

In order to circumvent the problem of uncertainty in the 9–12 month group, we have developed a severity index to function as a preliminary tool, allowing physicians to predict the disease course for a patient with onset between 9 and 12 months. The proposed index assesses patients for reflux, constipation, feeding difficulty, tracking difficulty, abnormal pupillary response, staring spells, clonus, seizures, hypertonia, and abnormal DTRs; the patient is scored a point for each symptom that is present. Ultimately, the results of our study suggest that patients with a score of 6–10 are expected to present with a severe, rapidly progressing infantile phenotype, while those with a score of ≤5 may continue to gain skills and are expected to present with a less severe and more slowly progressing infantile phenotype. However, because the index was developed retrospectively, it must be prospectively evaluated prior to becoming a standardized form of assessment. Additionally, while the current study included all patients with onset between 9 and 12 months in the index, regardless of their age at the time of their first evaluation, follow up analysis will attempt to identify a minimum and maximum age at baseline evaluation in which the index is most effective. These validation studies will require a larger cohort of patients.

Descriptions of Krabbe disease in the literature are pervaded by differences in nosology. Despite these tendencies, the phenotype for Krabbe disease exists on a continuum and classification into discrete categories is an arbitrary system imposed to help physicians better predict how a patient will present. Currently, the early-infantile phenotype is used to describe patients with onset between 0 and 5 months, and the definition for the late-infantile phenotype typically describes patients with onset between 6 months and 36 months. However, results of the current study bring these conventions into question and suggest that patients with onset between 6 and 12 months are more similar to patients with onset between 0 and 5 months than they are patients with onset after 12 months of age in respect to their symptomatology and rate and severity of progression. For example, a recent study investigating a cohort of early-infantile patients described 94% of patients as having feeding difficulties, 91% as having poor head control, 90% as having increased tone, and 82% as having fisted hands [13]. Similarly, of the 23 patients in our study with onset between 6 and 12 months, 87% had feeding difficulties, 88% had poor head control, 87% had increased tone, and 78% had fisted hands. In addition, a comparison of the 6–12 months cohort described in this study and a cohort of 85 patients with onset between 0 and 5 months, who were evaluated using the same prospective protocol as the current study, reveals a similar symptomatology and developmental trajectory for patients with onset between 0 and 12 months (unpublished data). Furthermore, because Krabbe disease is a rare condition, it is imperative that clinicians and researchers redefine phenotypic categories as our clinical knowledge of the disease advances.

**Fig. 6:**
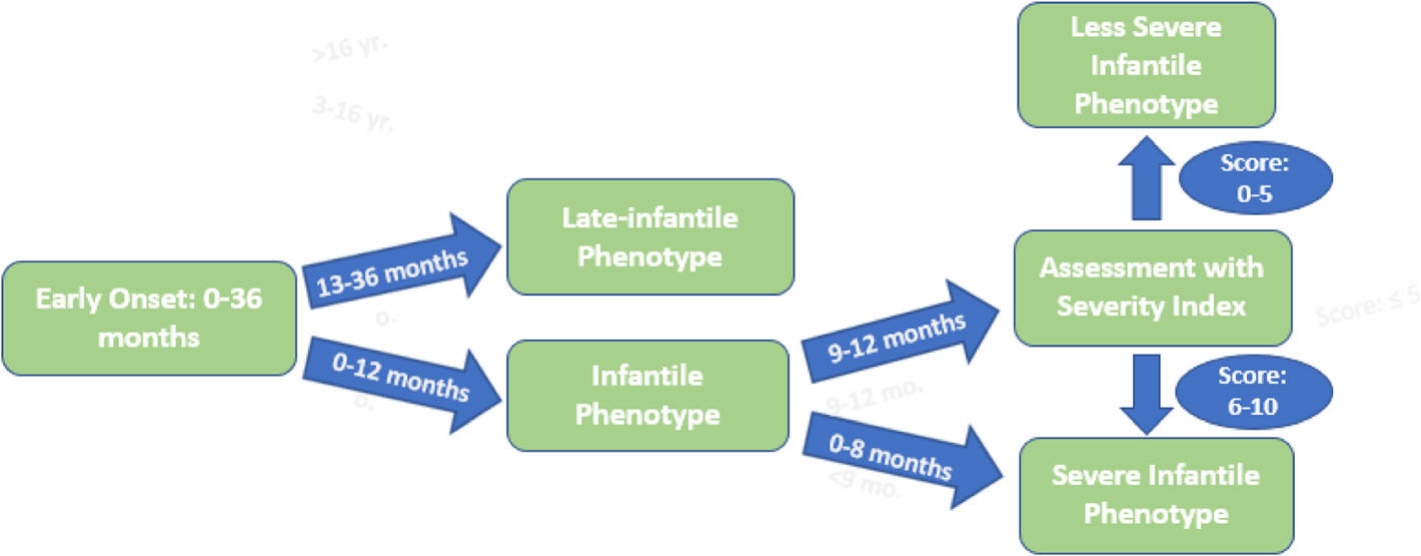
Classifying early onset Krabbe patients**.** This is a conceptual diagram of a proposed patient classification system for children with onset between 0 and 36 months of age. By using this classification system, physicians will be better able to predict patient phenotype. The text in the blue arrows represents age of onset. The text in the blue circles represent a patient’s score on the severity index. Patients can be classified as either “late-infantile” or “infantile.” Infantile patients are further subdivided into “severe infantile” and “less severe infantile” based on their age of onset and severity score

In consideration of these findings, we propose shifting the paradigm and classifying patients with onset between birth and 12 months as infantile, and patients with onset between 13 and 36 months as late-infantile. Although there was some variability in the 9–12 month cohort, most patients in the age range presented with a severe phenotype characterized by extreme irritability, feeding difficulties, gastroesophageal reflux, spasticity of lower extremities, and fisting, accompanied by axial hypotonia, rapid loss of acquired milestones, and staring episodes. Moreover, it is essential that physicians recognize the possibility that a patient with onset between 9 and 12 months may present with the severe infantile phenotype, as urgency and decisions to transplant are approached differently depending on the patient’s phenotype. This is especially important since outcomes after transplantation will differ depending on the rate and severity of disease progression, due to the fact that disease progression continues for several weeks until cells successfully engraft. Given these factors, we recommend that patients with onset between 9 and 12 months be referred to a specialized center and undergo a comprehensive evaluation immediately following their diagnosis. Once formally validated, the severity index will work in tandem with the new classification system (infantile = onset 0–12 months; late-infantile = onset 13–36 months) by providing a robust method for predicting the rate at which a patient with onset between 9 and 12 months will progress and offering a practical solution for the issue of phenotypic overlap between disease subtypes (Fig. 6).

In addition to allowing physicians to monitor patients for clinical indicators of the severe infantile phenotype, the severity index will enhance physicians’ abilities to select candidates that are likely to benefit from HSCT, which is especially important for ensuring early intervention in patients diagnosed through NBS. Because a lower severity scores indicates a less advanced disease state, a less severe phenotype, and slower disease progression, we believe that patients with a score of ≤5 are more likely to experience positive outcomes following HSCT than those who score 6–10. Although follow up studies are needed to substantiate this prediction, the claim reflects findings from other studies that report better treatment outcomes in asymptomatic and minimally symptomatic babies and children with less severe phenotypes [21–23]. While it is outside the scope of this study, it is important to mention that all transplanted patients with a score of ≤5 on the severity index have experienced better outcomes (unpublished data).

In addition to identifying differences between the ≤12 month and > 12 month group, another important finding from our study that differs from previous reports involves the results of neurophysiological testing. Previous neurophysiological studies in Krabbe disease report that VEP waveforms are normal in patients who have onset after 6 months, and only a small percentage of these patients have abnormal ABR, NCV, or EEG findings [19, 20]. However, the results of this study are remarkably different, with 42% (symptomatic, *n* = 8; asymptomatic, *n* = 1) of patients having abnormal VEP findings, 77% (symptomatic, *n* = 18; asymptomatic, *n* = 2) having abnormal ABR findings, 88% (symptomatic, *n* = 20; asymptomatic, *n* = 1) having abnormal NCV findings, and 61% (symptomatic, *n* = 10; asymptomatic, *n* = 1) having abnormal EEG findings. Since ABR, NCV, VEP, and EEG, as well as MRI, displayed evidence of disease progression in children who were clinically asymptomatic, the findings suggest that comprehensive neuroradiological and neurophysiological assessments can reveal manifestations of Krabbe disease in an asymptomatic infant earlier than the reported age of onset. This is an especially important finding for those asymptomatic children with low enzyme and private pathogenic variants that are being monitored for disease onset after diagnosis through NBS programs.

It is also worth noting that of the 35 patients included in our study, 26 (74%) were male and 9 (26%) were female; however, because Krabbe disease is inherited in an autosomal recessive manner, in theory, there should be an equal split between males and females, which suggests that there may be an underlying environmental or genetic mechanism contributing to the skewed distribution. Nevertheless, the unequal distribution has not been reported in any other studies and may be an artifact of the sample size. Further studies are therefore needed to determine if there is any significance to this finding.

Taken together, the data collected from children evaluated only once or longitudinally help to establish the initial symptoms and characteristics of disease progression, which can be beneficial in staging of the disease and predicting phenotype. Ultimately, a better understanding of the presenting symptoms of infantile and late-infantile Krabbe disease will increase awareness among pediatricians and result in earlier diagnostic referrals and recruitment for future clinical trials. The knowledge is necessary for monitoring disease progression and assessing the efficacy of therapeutic interventions. Such knowledge will become increasingly important as methodological and legislative advances in NBS practices continue. Future studies are needed to establish potential correlations between phenotype, GALC or psychosine levels and the rate and severity of disease progression, which will further improve clinicians’ ability to make decisions regarding the management and treatment of patients diagnosed via NBS.

Limitations of our study included restricted accessibility to some outside medical records and difficulty recruiting patients for longitudinal follow up once they became too ill to travel. In addition, children who underwent HSCT following their baseline visit were unavailable for longitudinal follow up. Considering asymptomatic and minimally symptomatic patients with less severe and/or slower progressing phenotypes are often the best candidates for HSCT, this may have caused a degree of implicit bias regarding the patients who were followed longitudinally. Furthermore, since the majority of patients with onset > 12 months were transplanted after their baseline visit, there is limited longitudinal data for this subgroup. Because certain symptoms were treated following baseline visits (i.e. reflux, feeding and swallowing difficulties, excretions, diarrhea, and constipation) the incidence of these symptoms in our population may be slightly altered in respect to the disease’s true natural history. Some data may also be subject to recall bias since a portion of the information was collected via a parent questionnaire. Nevertheless, the prospective design and standardized protocols for physical and neurodevelopmental evaluations make this study unique in providing a comprehensive description of the natural history of Krabbe disease.

## Conclusion

This study characterized the clinical features of Krabbe disease and advances our systematic understanding of the variability in phenotype for patients with onset between 6 and 36 months of life. In light of our findings, we propose reclassifying the group of patients with onset ≤12 month as infantile and the > 12 month group as late-infantile. These classifications will help ensure that asymptomatic or minimally symptomatic patients with a severe phenotype are treated with HSCT in a timely manner. Developing an improved understanding regarding the clinical course of Krabbe disease is essential to facilitating early diagnosis and assisting in the management and treatment of afflicted patients, especially in the context of NBS. There is a critical need for prospective natural history studies as they provide more rigorous and detailed descriptions of Krabbe disease and are crucial for determining appropriate endpoints in the design of successful clinical trials.

## Abbreviations

ABR: Auditory Brainstem Response
AE: Age-Equivalent
CSF: Cerebrospinal Fluid
DBS: Dry Blood Spot
DTR: Deep Tendon Reflexes
EEG: Electroencephalogram
GALC: Galactocerebrosidase
HGVS: Human Genome Variation Society
HSCT: Hematopoietic Stem Cell Transplantation
IRB: Institutional Review Board
MRI: Magnetic Resonance Imaging
NBS: Newborn Screening
NCV: Nerve Conduction Velocity
NDRD: Program for the Study of Neurodevelopment in Rare Disorders
VEP: Visual Evoked Potentials

## References

[1] Suzuki K (2003). Globoid cell leukodystrophy (Krabbe’s disease): update. J Child Neurol.

[2] Wenger DA, Rafi MA, Luzi P (2000). Krabbe disease: genetic aspects and progress toward therapy. Mol Genet Metab.

[3] Moser HW (2006). Peripheral nerve involvement in Krabbe disease: a guide to therapy selection and evaluation. Neurology.

[4] Castelvetri LC, Givogri MI, Zhu H (2011). Axonopathy is a compounding factor in the pathogenesis of Krabbe disease. Acta Neuropathol.

[5] Hagberg B, Sourander P, Svennerholm L (1963). Diagnosis of Krabbe’s infantile leucodystrophy. J Neurol Neurosurg Psychiatry.

[6] Sakai N (2009). Pathogenesis of leukodystrophy for Krabbe disease: molecular mechanism and clinical treatment. Brain Dev.

[7] Brodsky MC, Hunter JS (2011). Positional ocular flutter and thickened optic nerves as sentinel signs of Krabbe disease. J AAPOS.

[8] Morse LE, Rosman NP (2006). Myoclonic seizures in Krabbe disease: a unique presentation in late-onset type. Pediatr Neurol.

[9] Wenger DA, Suzuki K, Suzuki Y, Suzuki K, Scriver CR, Sly WS, Childs B (2001). Galactosylceramide lipidosis: globoid cell leukodystrophy (Krabbe disease). The metabolic and molecular bases of inherited disease.

[10] Duffner PK, Jalal K, Carter RL (2009). The Hunter's hope Krabbe family database. Pediatr Neurol.

[11] Duffner PK, Barczykowski A, Jalal K, Yan L, Kay DM, Carter RL (2011). Early infantile Krabbe disease: results of the world-wide Krabbe registry. Pediatr Neurol.

[12] Wenger DA, Escolar ML, Luzi P, Rafi MA (2013). Krabbe disease (globoid cell leukodystrophy). Scriver’s The Online Metabolic and Molecular Bases of Inherited Disease (OMMBID).

[13] Duffner PK, Barczykowski A, Kay DM, Jalal K, Yan L, Abdelhalim A, Gill S, Gill AL, Carter R (2012). Later onset phenotypes of Krabbe disease: results of the world-wide registry. Pediatr Neurol.

[14] Zhao S, Zhan X, Wang Y, Ye J, Han L, Qiu W, Gao X, Gu X, Zhang H (2018). Large-scale study of clinical and biochemical characteristics of Chinese patients diagnosed with Krabbe disease. Clin Genet.

[15] Shao Y, Choquet K, Piana RL (2016). B. Mutations in GALC cause late-onset Krabbe disease with predominant cerebellar ataxia. Neurogenetics.

[16] Wenger DA, Luzi P, Rafi MA (2014). Krabbe disease: are certain mutations disease-causing only when specific polymorphisms are present or when inherited in trans with specific second mutations?. Mol Genet Metab.

[17] Krabbe K (1916). A new familial, infantile form of diffuse brain-sclerosis. Brain.

[18] Hagberg B, Kollberg H, Sourander P, Akesson HO (1969). Infantile globoid cell leukodystrophy (Krabbe’s disease): a clinical and genetic study of 32 Swedish cases 1953-1967. Neuropadiatrie.

[19] Aldosari M, Altuwaijri M, Husain AM (2004). Brain-stem auditory and visual evoked potentials in children with Krabbe disease. Clin Neurophysiol.

[20] Husain AM, Altuwaijri M, Aldosari M (2004). Krabbe disease neurophysiologic studies and MRI correlations. Neurology.

[21] Escolar ML, Poe MD, Provenzale JM (2005). Transplantation of umbilical-cord blood in babies with infantile Krabbe’s disease. New Engl J Med.

[22] Lim ZY, Ho AY, Abrahams S (2008). Sustained neurological improvement following reduced-intensity conditioning allogenic hematopoietic stem cell transplantation for late-onset Krabbe disease. Bone Marrow Transpl.

[23] Escolar ML, Yelin K, Poe MD (2006). Neurodevelopmental outcomes of children with infantile Krabbe disease treated with umbilical cord blood transplantation: 10 years of follow up. CML: Lysosomal Storage Disease.

[24] Escolar ML, Poe MD, Martin HR, Kurtzberg J (2006). A staging system for infantile Krabbe disease to predict outcome after unrelated umbilical cord blood transplantation. Pediatrics.

[25] Lantos JD (2011). Dangerous and expensive screening and treatment for rare childhood diseases: the case of krabbe disease. Dev Disabil Res Rev.

[26] Orsini JJ, Saavedra-Matiz CA, Gelb MH, Caggana M (2016). Newborn screening for Krabbe's disease. J Neurosci Res.

[27] Wasserstein MP, Andriola M, Arnold G, Aron A, Duffner P, Erbe RW, Escolar ML, Estrella L, Galvin-Parton P, Iglesias A, Kay DM (2016). Clinical outcomes of children with abnormal newborn screening results for Krabbe disease in New York state. Genet Med.

[28] Kwon JM, Matern D, Kurtzberg J, Wrabetz L, Gelb MH, Wenger DA, Ficicioglu C, Waldman AT, Burton BK, Hopkins PV, Orsini JJ (2018). Consensus guidelines for newborn screening, diagnosis and treatment of infantile Krabbe disease. Orphanet J Rare Dis.

[29] Escolar ML, Kiely B, Shawgo (2017). Psychosine, a marker of Krabbe phenotype and treatment effect. Mol Genet Metab.

[30] Biffi A, Aubourg P, Cartier N (2011). Gene therapy for leukodystrophies. Hum Mol Genet.

[31] Matthes F, Andersson C, Stein A (2015). Enzyme replacement therapy of a novel humanized mouse model of globoid cell leukodystrophy. Exp Neurol.

[32] Martin HR, Poe MD, Reinhartsen D (2008). Methods for assessing neurodevelopment in lysosomal storage diseases and related disorders: a multidisciplinary perspective. Acta Paediatr Suppl.

[33] Bruininks RH, Woodcock RW, Weatherman RF, Hill BK (1996). Scales of independent behavior-revised.

[34] Folio MR, Fewell RR. Peabody developmental motor scales, 2nd ed. Austin: Pro-Ed; 2000.

[35] Mullen EM (1995). Mullen scales of early learning AGS edition.

[36] Russell DJ, Rosenbaum PL, Wright M, Avery LM. Gross motor function measure (GMFM-66 and GMFM-88) User's manual. 2nd ed. London: Mac Keith Press; 2013.

[37] Burger-Caplan R, Saulnier CA, Sparrow SS. Vineland adaptive behavior scales. InEncyclopedia of clinical neuropsychology: Springer International Publishing; 2018. p. 1–5.

[38] Kuczmarski RJ, Ogden CL, Guo SS (2002). 2000 CDC growth charts for the United States: methods and development. National Center for Health Statistics Vital Health Stat.

[39] den Dunnen JT (2017). Describing sequence variants using HGVS nomenclature. InGenotyping.

